# Influence of pollen limitation and inbreeding depression in the maintenance of incomplete dichogamy in *Salvia elegans*

**DOI:** 10.1002/ece3.2827

**Published:** 2017-04-27

**Authors:** Víctor Rosas‐Guerrero, Diego Hernández, Eduardo Cuevas

**Affiliations:** ^1^ Facultad en Desarrollo SustentableCampus Costa GrandeUniversidad Autónoma de GuerreroTecpan de GaleanaGuerreroMéxico; ^2^ Facultad de BiologíaUniversidad Michoacana de San Nicolás de HidalgoMoreliaMichoacánMéxico

**Keywords:** herkogamy, Lamiaceae, pollen viability, protandry, sexual interference, stigma receptivity

## Abstract

The widespread presence of incomplete dichogamy (i.e., partial separation in time between male and female phases) in flowering plants is a long‐standing question in floral evolution. In this study, we proposed four scenarios in which depending on the particular combination of pollen limitation and inbreeding depression, the presence of complete dichogamy, incomplete dichogamy, or adichogamy may be favored. Moreover, we evaluated the role of pollen limitation and inbreeding depression in a natural population of *Salvia elegans* to test the validity of our predicted scenarios. Our results indicate that *S. elegans* is partially protandrous as pollen viability and stigma receptivity overlap in the last days of life of the flower. Furthermore, through pollination treatments, we found no evidence of pollen limitation or inbreeding depression in any of the evaluated fitness components. As expected by one of the proposed scenarios, incomplete dichogamy seems to be favored in plants with absence of inbreeding depression and pollen limitation as a way to diminish interference between male and female functions.

## 1 Introduction

Temporal separation of male and female phases (dichogamy) within a plant is an extremely common floral feature in hermaphrodite species (Bertin, 1993; Bertin & Newman, 1993; Cruden, 1988). Two forms of dichogamy are recognized: protandry, in which anthers dehisce before the stigma becomes receptive; and protogyny, in which the stigma is receptive before the pollen is released. Sexual phases could be completely separated (complete dichogamy), completely overlapped (adichogamy), or partially overlapped (incomplete dichogamy; Lloyd & Webb, 1986; Stout, 1928).

Given the expected reduction in self‐pollination, dichogamy has been invoked as a mechanism for avoiding inbreeding depression (Bertin & Newman, 1993; Darwin, 1876; Lloyd & Webb, 1986; Ornduff, 1969; Sargent, Mandegar, & Otto, 2006; Stout, 1928), that is, the reduction in fitness and vigor of inbred relative to outbreed progeny. However, reviews of empirical data of dichogamous species arise an apparent redundancy of function: Many dichogamous plant species also present a self‐incompatibility system (a mechanism supposed to also prevent self‐pollination; Bertin & Newman, 1993; Routley, Bertin, & Husband, 2004). Moreover, some studies have found no support for high inbreeding depression in dichogamous plant species (e.g., Hossaert‐McKey & Bronstein, 2001). These results have strengthened the alternative explanation that dichogamy could evolve to reduce interference between male and female functions (Bertin, 1993; Holsinger, Feldman, & Christiansen, 1984; Lloyd & Webb, 1986; Lloyd & Yates, 1982; van der Pijl, 1978; Wyatt, 1983), which has been supported by empirical evidence (Cesaro et al., 2004; Dai & Galloway, 2011; Lloyd & Yates, 1982; Routley & Husband, 2003).

Even when avoidance of inbreeding depression or sexual interference could help to explain the evolution of temporal separation of sexual phases, none of these hypotheses by itself is sufficient to explain why so many plant species display incomplete dichogamy, as either self‐fertilization or sexual interference may occur given the partial overlap of both sexual functions. Even when some studies have investigated potential causes that might affect the duration of female and male phases in dichogamous plants (e.g., Devlin & Stephenson, 1984; Koptur et al., 1990; Richardson & Stephenson, 1989; Sargent & Roitberg, 2000; Temeles & Pan, 2002), there is a gap of theoretical (but see Sargent et al., 2006) or empirical studies of the possible causes of the widespread occurrence of incomplete dichogamy in flowering plants.

In this study, we proposed that a particular combination of pollen limitation (i.e., an inadequate quantity or quality of pollen resulting in a reduction in fruit and/or seed production in plants) and inbreeding depression could help to explain the maintenance of incomplete dichogamy, complete dichogamy, or complete adichogamy. Furthermore, we tested the role of these selective forces in maintaining temporal separation of male and female phases in *Salvia elegans*, a self‐compatible, hummingbird‐pollinated plant with flowers that last for 4 days (Espino‐Espino, Rosas, & Cuevas‐García, 2014). Specifically, we aimed to: (1) determine pollen viability and stigma receptivity throughout all flower lifespan to define temporal overlap of sexual phases; (2) estimate pollen limitation through natural and manual pollinations; and (3) estimate inbreeding depression at three life stages.

Whereas inbreeding depression is expected to favor complete dichogamy (Bertin & Newman, 1993), pollen limitation is expected to favor gender overlap to maximize self‐fertilization (Knight et al., 2005). Indeed, it has been proposed that even in the presence of some inbreeding depression, selfing could be selected as a mechanism for reproductive assurance under conditions of pollen limitation (Goodwillie, Kalisz, & Eckert, 2005; Kalisz, Vogler, & Hanley, 2004). Accordingly, we expect that incomplete dichogamy should be favored in plants that experience inbreeding depression but also strong pollen limitation. Alternatively, we expect that complete dichogamy should be selected in plants with strong inbreeding depression but no pollen limitation, whereas complete adichogamy should be expected in plants without inbreeding depression but strong pollen limitation. Finally, we expect that incomplete dichogamy should also be favored in plants without neither strong inbreeding depression nor pollen limitation as a way to minimize pollen–pistil interference (Table 1). Even when outbreeding depression may have some role in the maintenance of dichogamy, we did not considered it on these scenarios as its occurrence apparently is much less common than inbreeding depression.

**Table 1:** Expected scenarios of temporal separation of sexual phases in flowering plants depending on the presence/absence of pollen limitation and inbreeding depression

	Pollen limitation	
	Presence	Absence
**Inbreeding depression**		
**Presence**	Incomplete dichogamy	Complete dichogamy
**Absence**	Complete adichogamy	Incomplete dichogamy

See Section 1 for a detailed explanation of each scenario.

Given the presence of self‐compatibility in *S. elegans* (Espino‐Espino et al., 2014), the relatively high plant density (0.8 individuals/m^2^), and the active presence of hummingbirds at the study site (Espino‐Espino, 2012), we expect an absence of inbreeding depression, as well as low levels of pollen limitation. Therefore, according to previous scenarios, we predict that this species will display incomplete dichogamy.

## 2 Materials and Methods

### 2.1 Study species and study site

**Figure 1:**
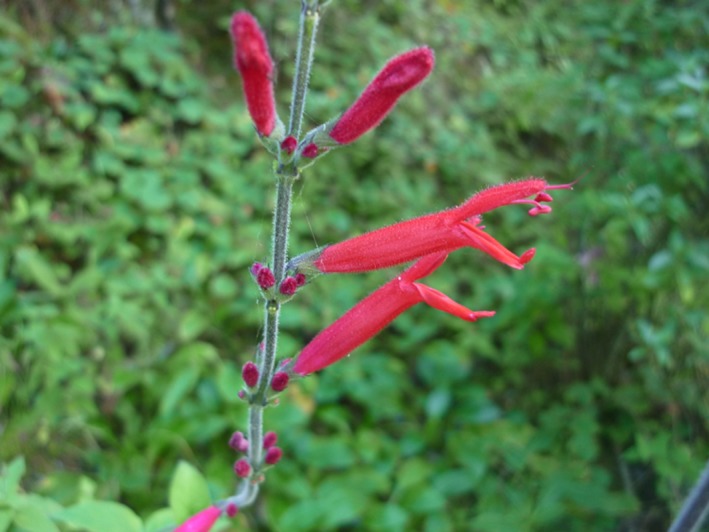
The tubular red flowers of *Salvia elegans*, showing the exerted stigma and the two stamens

The genus *Salvia* (Lamiaceae) comprises more than 900 plant species distributed in Europe, Asia, and America (Walker, Sytsma, Treutlein, & Wink, 2004). *Salvia elegans* is an endemic shrub of Mexico with bilabiate tubular red flowers, suggesting an ornithophilous pollination syndrome (Figure 1; Rzedowski & Rzedowski, 2005). Consistent with this expectation, Lara (2006) and Espino‐Espino et al. (2014), found that their flowers are visited by several hummingbird species in some populations in the state of Tlaxcala and Michoacan, Mexico, respectively. As all members of this genus, each flower has four ovules.

In the studied population, *Salvia elegans* produce flowers from October to April, with a flowering peak in January (Hernández‐Pérez, 2009). Nectar volume varies from 2.4 ± 0.39 μl (mean ± *SE*, here and hereafter) in one‐day‐old flowers to 5.3 ± 0.62 μl in three‐day‐old flowers, whereas average nectar concentration remains relatively constant around 33% throughout lifespan (E. Cuevas, unpublished data).

The study was carried out at the protected natural area of “Cerro Garnica” with an extent of 968 ha located 50 km east of Morelia city, in the state of Michoacan, Mexico. The Cerro Garnica reserve (19°40′04″N; 100°49′36″W) belongs to the Trans‐Mexican Volcanic Belt and has an average altitude of 2,950 masl and a temperate subhumid climate (mean annual temperature 12–14°C and annual rainfall 1,200–1,400 mm; Romero, 1991). The vegetation is pine–oak forest with predominance of *Quercus laurina*,* Quercus crassipes*,* Pinus pseudostrobus*,* Pinus teocote*, and *Abies religiosa* (Romero, 1991).

### 2.2 Pollen grain viability and stigma receptivity

Pollen viability was determined from two bagged flowers of 12 plants per day during 4 days (*n* = 96 flowers). In the laboratory, 300–400 pollen grains per anther were observed under a microscope. Pollen grains were stained using Alexander's stain, and viable (intense dyed) and nonviable grains (weak dyed) were counted (Kearns & Inouye, 1993).

Stigma receptivity was determined by manual outcross pollinations on three flowers (enclosed with mesh bags before flower aperture) from 12 plants per day during 4 days (*n* = 144 flowers). Pollen from a flower of another plant at least 10 m away was deposited on the stigma of one‐, two‐, three‐, or four‐day‐old focal flowers and then re‐bagged until corolla abscission. All flowers used as pollen donors were recently open and full of pollen. Even though there is some possibility that pollen from some plants were of “poor quality,” we did not detect an effect of plant factor on the evaluated reproductive components, suggesting all plants used had more or less the same pollen quality (see Section 3). Pollen viability and stigma receptivity were evaluated between 09:00 and 11:00 hr. Approximately 1 month later, mature fruits were collected whereas developed seeds were counted.

The proportion of viable pollen grains from flowers of different ages were compared using a generalized linear mixed model (GLMM), with the proportion of viable pollen grains (binomial distribution) as dependent variable, flower age as fixed effect, and plant as a random factor.

The stigma receptivity from flowers of different ages was analyzed using a GLMM, by comparing the fruit set (binomial distribution) and developed seed number (Poisson distribution and log function) as dependent variables, flowering day as a fixed effect, and plant as a random factor. When a significant difference was found, a Tukey's test was used to determine in which day the fruit set or seed number was greater.

### 2.3 Pollen limitation

Pollen limitation was estimated by comparing the fruit set and number of developed seeds per fruit under open pollination and manually outcrossed pollination. For open pollination, 20 flowers of each of 15 plants were left uncovered to allow natural pollination. For manually outcrossed pollination, 20 flowers (of three‐ or four‐day‐old flowers, given their higher stigma receptivity; see Section 3) of each of another 15 plants were hand‐pollinated with pollen from a different plant separated at least 10 m from focal flowers. Around 90% of flowers per day per plant were used for both treatments. Fruit set and number of developed seeds per fruit under open pollination and manually outcrossed pollination were compared by means of a GLMM with fruit set (binomial distribution) or developed seed number (Poisson distribution) as dependent variables, treatment (open pollination vs. manually outcrossed) as fixed effect, and plant as a random factor. As the variance explained by plant was insignificant, this factor was removed from the model.

### 2.4 Inbreeding depression

Inbreeding depression was estimated as recommended by Ågren and Schemske (1993) through the calculation of the “relative performance of crosstypes” (RP) by computing: δ = 1 − ws/wo, when ws ≤ wo; or δ = wo/ws − 1, when ws > wo, where ws and wo are the mean fitnesses of selfed and manually outcrossed progeny per plant, respectively. This index varies from −1, indicating outbreeding depression, to 1, which reflect inbreeding depression, whereas values near 0 reflect absence of depression. RP was calculated for four fitness components: (1) fruit set; (2) seed number per fruit; (3) seed mass; and (4) seed germination. Selfed progeny was obtained from manual self‐pollination, where each flower was hand‐pollinated with pollen from the same plant (*n* = 80 flowers from 12 plants), whereas outcrossed progeny was obtained as explained above (*n* = 82 flowers from 12 plants). All manual pollinations were carried out on three‐ or four‐day‐old flowers. Mature fruits were collected approximately 4 weeks after pollination and then fruit set, seed production (*n* = 62 self‐ and 52 cross‐fertilized fruits) and seed mass (*n* = 58 self‐ and 52 cross‐fertilized seeds) were calculated. A random sample of seeds produced by selfing and manually outcrossed pollinations (*n* = 100 seeds from 11 plants and 94 seeds from 12 plants, respectively) was planted and grown in a greenhouse. Seeds were planted in 10‐cm‐diameter pots, and germination rate was evaluated by determining the proportion of seedlings with cotyledons at 3 weeks after seed planting.

Selfed and manually outcrossed progeny from all fitness components were compared by means of GLMM, with fruit set (binomial distribution), seed number (Poisson distribution) seed mass (Gaussian distribution) or germination rate (binomial distribution) as dependent variables. Each trait was analyzed separately. Treatment (manually selfed vs. manually outcrossed) was included as fixed effect and plant as a random factor. For each fitness component, we used a one‐sample *t* test to determine whether RP deviated significantly from 0. All statistical analyses were performed using R 2.14.1 (R Development Core Team 2010).

## 3 Results

### 3.1 Pollen grain viability and stigma receptivity

**Figure 2:**
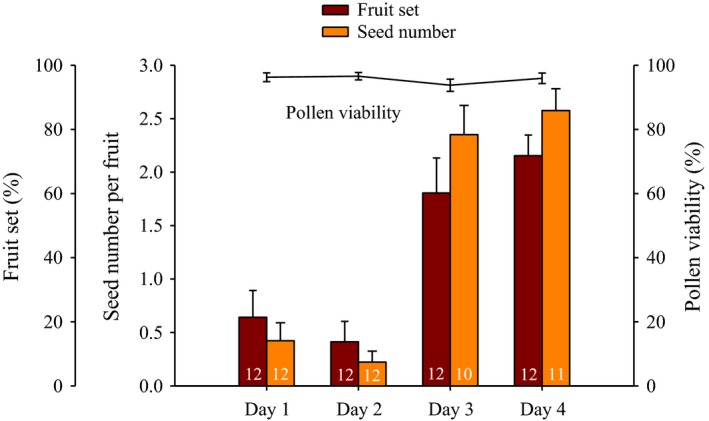
Average (±*SE*) pollen viability (solid line; *n* = 24 flowers per day, 300–400 pollen grains per anther) and stigma receptivity (numbers inside bars indicate sample sizes) of *Salvia elegans* during all flower lifespan. Stigma receptivity was estimated as fruit set and seed number per fruit per day from manually outcrossed pollination treatments. No differences were detected in pollen viability with respect to flower age, whereas stigma receptivity was greater at three‐ or four‐day‐old flowers than at one‐ or two‐day‐old flowers in both fruit set and seed number per fruit after a generalized linear mixed model

Pollen viability and stigma receptivity tests indicate that *S. elegans* display incomplete protandry as pollen was viable before the stigma became receptive on the first 2 days but a considerable overlap occurred between both stages at the third and fourth day (Figure 2). High percentages of pollen viability were obtained, ranging from 94% (±*SE* = 9.3) at three‐day‐old flowers to 97% (±1.1) at two‐day‐old flowers. No differences were detected in pollen viability with respect to flower age (*Z*_3,92_ = 2.49, *p *=* *.91).

Fruit set obtained by manual outcross pollination ranges from 13.7 (±6.37) at two‐day‐old flowers to 71.8 (±6.42) at four‐day‐old flowers and differ significantly among flower ages (*Z*_3,140_ = 2.12, *p *=* *.03; Figure 2). In a similar way, the mean number of developed seeds per fruit varied significantly depending on flower age (from 0.2 to 2.6; *Z*_3,158_ = −3.13; *p *<* *.001), being higher in three‐ or four‐day‐old flowers than in one‐ or two‐day‐old flowers.

### 3.2 Pollen limitation

**Figure 3:**
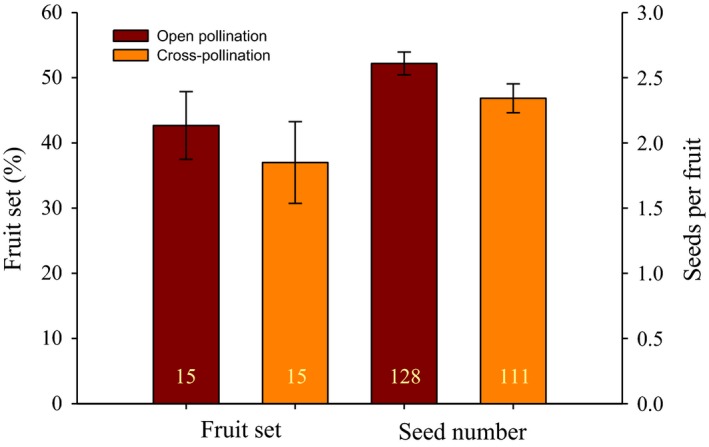
Pollen limitation in *Salvia elegans*. Fruit set and number of seeds per fruit between open and manually outcrossed pollination (mean ± *SE*). Numbers inside bars indicate sample size: number of plants for fruit set (20 flowers per plant) and number of fruits for seed number

Similar values of fitness components on open and manual outcross pollination treatments indicated no evidence of pollen limitation in the studied population. Average fruit set of manually outcrossed flowers (37.0 ± *SE* = 6.26) did not differ from flowers with open pollination (42.7 ± 5.21; *Z*_1,29_ = −0.311, *p *=* *.75; Figure 3). Similarly, the mean number of seeds per fruit obtained from manually outcrossed pollinations (2.3 ± 0.11) did not differ from that of open‐pollinated flowers (2.6 ± 0.09; *Z*_1,237_ = −1.3, *p *=* *.19; Figure 3).

### 3.3 Inbreeding depression

We found no evidence of inbreeding depression in any of the evaluated fitness components. The average fruit set (*Z*_1,22_ = 0.66, *p *=* *.5), seed number (*Z*_1,112_ = 0.258, *p *<* *.79), seed mass (*Z*_1,108_ = 0.052, *p *=* *.95), and seed germination (*Z*_1,21_ = −0.47, *p *=* *.63) between self and outcross treatments did not differ statistically (Table 2). Similarly, the RP estimates of fruit set, seed number, seed mass, and germination rate (−0.20, −0.01, −0.11, and −0.22, respectively) did not deviate significantly from 0 (*p *>* *.05 in all cases), indicating a lack of inbreeding depression. Given the lack of self‐incompatibility in this plant species (Espino‐Espino et al., 2014), the effects of inbreeding depression on fitness traits considered in this study should not be confounded with partial self‐incompatibility effects.

**Table 2:** Mean of progeny fitness components after manual self and manual outcross pollinations and relative performance (RP) as an estimation of inbreeding depression in a natural population of *Salvia elegans*

Fitness component	Self (±*SE*) [*n*]	Outcross (±*SE*) [*n*]	Mean RP (*p*)
Fruit set (%)	79.3 (5.17) [12]	63.0 (7.67) [12]	−0.20 (.06)
Seed number	2.6 (0.14) [62]	2.5 (0.13) [52]	−0.01 (.86)
Seed mass (μg)	2.4 (0.16) [58]	1.96 (0.16) [52]	−0.11 (.21)
Seed germination (%)	64.4 (4.32) [11]	54.6 (8.85) [12]	−0.22 (.15)

*p* Values after a two‐tailed *t* test for difference between mean RP and 0 are shown.

## 4 Discussion

Our study represents the first one that explores simultaneously the role of inbreeding depression and pollen limitation in the maintenance of incomplete dichogamy. We detected incomplete dichogamy in *S. elegans* and no evidence of inbreeding depression or pollen limitation. As expected by one of our four predicted scenarios (see Table 1), incomplete dichogamy was favored when there is an absence of inbreeding depression and a lack of pollen limitation as a way to diminish interference between male and female functions.

One of the few ideas that try to explain the evolution of incomplete dichogamy was suggested by Sargent et al. (2006). They proposed that even when dichogamy is strongly favored either by anther–stigma interference or inbreeding depression, a substantial overlap should be selected between the timing of pollen availability and stigma receptivity to ensure pollination. We suggest that this overlap should be favored even more when pollen limitation prevail in the population.

Our results indicate that there is no evidence of pollen limitation in *S. elegans*; thus, complete dichogamy should be expected to maximize cross‐pollination. However, given the lack of inbreeding depression found in this species, self‐pollination could be a reliable mechanism to ensure reproduction if mates are scarce. Thus, incomplete dichogamy should be favored instead of complete dichogamy.

Even when *S. elegans* is self‐compatible and displays a substantial overlap between anther dehiscence and stigma receptivity on three‐ and four‐day‐old flowers, autonomous selfing did not occur (Hernández‐Pérez, 2009). This is in agreement with the results obtained by Bertin and Newman (1993) who found that nonautogamous species exhibit dichogamy more often than species capable of selfing without a pollen vector. The lack of autonomous selfing in *S. elegans* may be due to its approach herkogamy (i.e., the stigma is located above the anthers; see Figure 1), avoiding the contact between anthers and stigma. Consequently, this plant species requires of pollinators to ensure seed production either through autogamy (i.e., pollination of a flower by its own pollen), geitonogamy (i.e., pollination between flowers of the same individual), or allogamy (i.e., pollination between flowers from different individuals).

The lack of pollen limitation and the dependence of pollinators to set seeds suggest that pollinators were not scarce in our study site, at least in the year of study. Routley et al. (2004) suggest that pollinator availability can alter the form of dichogamy. Even in 19th century, Müller (1883) observe that dichogamy is more common in species with frequent insect visits than in those with few insect visits. Indeed, Brys, Geens, Beeckman, and Jacquemyn (2013), found that plants from pollinator‐poor environments showed a significant reduction in the level of dichogamy. Similarly, Zhang, Zhao, and Wang (2011) found that when pollinators were scarce, the male phase in *Glechoma longituba* was larger allowing overlap of sexual phases and facilitating self‐pollination as a reproductive assurance. Accordingly, it is expected that dichogamy should be maintained in this plant species if pollinator availability remains constant through years. To corroborate this expectation, it is necessary to evaluate more populations of *S. elegans* with different environmental conditions to evaluate the level of plasticity in dichogamy.

The absence of inbreeding depression on the evaluated components on *S. elegans* suggest the effect of previous genetic purge promoted by continuous selfing as inbred individuals harboring deleterious alleles may die or not reproduce, removing these alleles from the population (Crnokrak & Barrett, 2002). Consistent with this idea, Husband and Schemske (1996) found that the average magnitude of inbreeding depression in predominantly selfing species (δ = 23) was less than half of that in predominantly outcrossing species (δ = 53). If is true that *S. elegans* has experienced continuous selfing episodes for a long time, this plant species may have not suffered from pollinator limitation in the long term, given that selfing depends on pollinators. The absence of pollinator limitation found in this study agrees with this assumption.

Theory predicts that selfing is favored when values of inbreeding depression (or RP values) were less than 0.5 (Charlesworth & Charlesworth, 1987). Given that all RP values estimated for *S. elegans* were far less than 0.5, it seems that selfing occur frequently in this population, as mentioned above. Thus, it seems that protandry is an inefficient mechanism to avoid selfing, as some authors previously suggested (e.g., Bertin & Newman, 1993; Routley et al., 2004). If this is true, protandry seems to remain in this population to reduce interference between male and female functions, whereas incomplete dichogamy seems to be maintained to ensure reproduction when mates are scarce.

As in several studies (e.g., Dai & Galloway, 2011; Routley & Husband, 2003), dichogamy has been proposed to be under selection to reduce sexual interference in plants, even in the absence of inbreeding depression (Sargent et al., 2006). Therefore, the incomplete protandry found in this species should increase the probability of pollen grains to be exported and enhance male reproductive success (but see Routley & Husband, 2006). Further analyses with molecular markers could help to elucidate this idea.

The results stemming from our study suggest that inbreeding depression, pollinator limitation, and self‐interference avoidance may have played an important role in the maintenance of incomplete dichogamy. However, even when our experimental manipulations detected no differences on fruit set, seed number, seed mass, or germination rates between manual self and outcross treatments, inbreeding depression could be expressed in postgermination stages not evaluated in the present study, as in the majority of predominantly selfing species, the expression of inbreeding depression may occur late in the life cycle (Husband & Schemske, 1996). Moreover, pollen limitation may vary among years if pollinator visitation vary temporarily; therefore, a long‐term study of variation in pollen limitation is required to explore their consistency as selective force in floral evolution.

Further experiments should explore the visitation rate of pollinators in the different stages of the flower of *S. elegans* to evaluate their role in self‐pollination and geitonogamy, as well as their relationship with the schedule of floral rewards. Finally, several case studies should be evaluated to test the reliability of the four scenarios proposed in this study (see Table 1) to get a better understanding of the evolution of incomplete dichogamy. Currently, we are performing a review of available studies to test the validity of these assumptions.

## Conflict of Interest

The authors have no conflict of interests to declare.
